# TDMA-Based Dual-Mode Communication for Mobile Wireless Sensor Networks

**DOI:** 10.3390/s121216194

**Published:** 2012-11-22

**Authors:** Ankur Mehta, Branko Kerkez, Steven D. Glaser, Kristofer S. J. Pister

**Affiliations:** 1 Department of Electrical Engineering and Computer Sciences, UC Berkeley, Berkeley, CA 94720, USA; E-Mail: pister@eecs.berkeley.edu; 2 Department of Civil and Environmental Engineering, UC Berkeley, Berkeley, CA 94720, USA; E-Mails: bkerkez@berkeley.edu (B.K.); glaser@berkeley.edu (S.D.G.)

**Keywords:** mobile wireless sensor networks, WSN, MAV, TDMA, IEEE 802.15.4

## Abstract

Small highly mobile robots, and in particular micro air vehicles (MAVs), are well suited to the task of exploring unknown indoor environments such as buildings and caves. Such a task imposes a number of requirements on the underlying communication infrastructure, with differing goals during various stages of the mission. This work addresses those requirements with a hybrid communications infrastructure consisting of a stationary mesh network along with the mobile nodes. The combined network operates in two independent modes, coupling a highly efficient, low duty cycle, low throughput mode for routing and persistent sensing with a burst mode for high data rate communication. By strategically distributing available frequency channels between the mobile agents and the stationary nodes, the overall network provides reliable long-term communication paths while maximizing data throughput when needed.

## 1. Introduction

Small, cheap, highly mobile robots can be used to solve a wide variety of problems [1]. The availability of off-the-shelf components for such micro air vehicles (MAVs) has made them an active topic of current research and even commercial development (e.g., [2–4]). In particular, MAVs are well suited for exploration of unknown or difficult to access indoor environments, such as buildings and caves. They can be used to map out a space of interest, or search within for items of interest. MAVs can also be used to deploy a stationary wireless sensor network (WSN) for long-term persistent sensing of that environment.

Though there are many details differentiating specific mission scenarios, at the most basic they all involve gathering data throughout an unknown environment and passing it to a remote base station. Only in a very narrow range of specifications can such a mission be accomplished without any communication at all (e.g., by storing data on board then physically returning to the base station); otherwise, multihop wireless network design becomes an integral consideration in such missions. Different mission parameters present different tradeoffs, and there may be no single network design that will satisfy all MAV requirements. However, there are a number common traits, which leads to a general framework that can be used as a starting point for developing a ubiquitous MAV network architecture.

In this paper we present a design for a network infrastructure that can accommodate typical MAV missions. By building on existing solutions for the various subproblems, we can create a hybrid network system that can effectively operate in a mode appropriate to the task at hand. In Section 2 we will describe typical MAV missions, breaking it up into common subproblems. A description of previous research, along with hardware used for such missions will also be included. The communications requirements inherent to a MAV will be discussed in Section 3, followed by the state of the art protocol for communications in a wireless mesh network in Section 4. These two modes will be combined into a single hybrid protocol, as described in Section 5. Finally, we will identify areas where this implementation is still insufficient or unclear, and propose various solutions to complete the design in Section 6. We will close with a discussion of future research needs to properly design a network infrastructure to support MAV missions.

## 2. Background

### 2.1. Missions and Requirements

**Figure 1.**
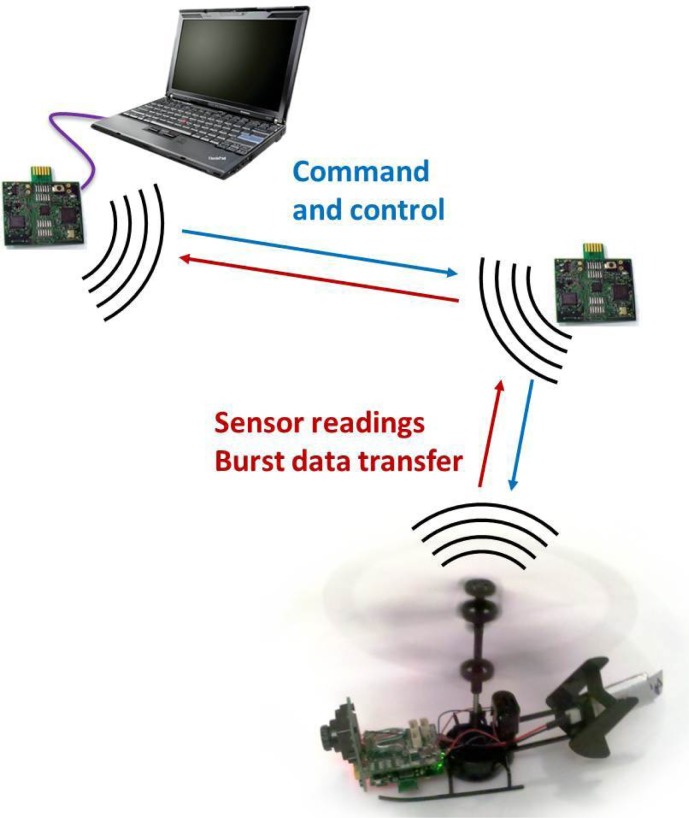
This schematic shows a deployed wireless sensor network infrastructure for a MAV mission. The laptop represents a base station and acts as a network manager, coordinating communications while serving as a sink for data generated by the MAV and sensor mesh. Data generated by the MAV is sent through the multihop mesh.

A major motivating example of the need for MAV networks is shown in Figure 1. In this example, a MAV has dropped a number of nodes in an unknown environment, and is wirelessly communicating with a base station over the resulting stationary infrastructure. A star topology on the underlying network would limit communication to only those nodes a single hop from the base station, while a multihop tree topology would be susceptible to loss of connectivity from the failure of a single node. As such, the network must form a multihop mesh topology, where nodes can have multiple neighbors, and data can pass through several nodes on their way to and from the base station.

These stationary network nodes serve the purpose of relaying information between the MAV agent and the base station, while also acting as a long-term persistent sensing platform, which can be left in place indefinitely to collect information about its environment. The challenge of designing such a system thus lies in adapting the wireless network to both long-term, low data rate reliable sensing missions, while accommodating the burst of short-term, high data rate transmissions of MAV agents.

The specific MAV mission will define the tradeoffs necessary to design its underlying network. However, many of those design decisions can be accomplished by tuning various parameters in a general framework. At its most general, a MAV mission has the following components:

- Navigate through the environment
- Deploy sensors and repeater nodes
- Relay data back from MAV and deployed sensors

The arbitrary topology of an unknown environment means that line-of-sight communication from the MAV to the outside world is often impossible. Thus, the MAV must carry along and deploy a payload of repeater nodes along its flight path. These drop-off nodes must establish a wireless network over which the MAV can communicate to an external base station.

The objective of the general MAV mission is to relay data from inside the unknown environment to the outside world. This data can come from the sensors on the MAV, using the deployed network nodes merely as a communications infrastructure, or it can also be generated by sensors on the deployed nodes themselves. In the case of reconnaissance-type missions, data from MAV sensors will be composed of short, high volume bursts (camera feeds, microphones, *etc.*). The stationary network nodes will generate low rate data over the long-term for purposes of anomaly detection or the monitoring of slowly-changing environmental phenomena (footstep detection, smoke detection, *etc*.).

The most significant design constraints in such missions are weight and power consumption; the two go hand-in-hand. The lower the mass of the MAV with its payload, the less power is needed to fly, thus permitting longer missions. Similarly, the lower the power consumption, the smaller and lighter the battery needed for a given mission. Since the deployed network is initially dead payload weight on the MAV, minimizing overall network power consumption minimizes the battery requirements on the dropped-off nodes (or eliminates in entirely in favor of energy scavenging sources), thus lightening the load and increasing the scope of possible missions.

Guidance of the MAV can either be handled remotely, via a central base station, or autonomously through on-board processing. Remote control simplifies flight processing by offloading guidance and navigation decisions to a central operator. However, it imposes high throughput and low latency requirements on the network. On-board data must be passed rapidly to the operator to facilitate real-time control of the MAV. Due to possible intra-network radio interference and limited centralized radio resources, there exist limits on the ability to utilize multiple MAVs during a mission. Autonomous MAV operation, on the other hand, significantly increases on-board sensing and processing requirements, but reduces required sustained data rates. Autonomous operation can also enable multiple MAVs in a single network, which increases complexity of the network routing and general network resource allocation.

Given such tradeoffs, there are a number of important parameters to consider when evaluating a MAV system. The potential capability of the system is directly related to the mission duration, which in turn depends on power consumption. The efficacy at which the MAV can accomplish its goals is directly related to the data transfer capabilities within the system, which can be measured both by data throughput and network latency.

### 2.2. Prior Work

The problem of maintaining reliable communication to mobile nodes through a WSN has been studied both generally and for specific mission scenarios. In such studies, especially those dealing with MAVs, communication was often assumed to be single-hop line-of-sight from the mobile node to the base station [5,6]. As previously described, most missions which have data generated in RF-challenged environments are simply infeasible without multihop communication to the outside world. Furthermore, lack of frequency channel diversity can cause a selected transmission channel to perform poorly due to external interference or multipath fading [7]. Even brief drops in connectivity due to such interference can have severe adverse real-time performance effects during a MAV mission.

Alternately, if relaying MAV data over a stationary multihop mesh network, nodes in the network are required to be always on – when not transmitting, each node must keep its radio in receive mode to ensure that data from the mobile node will be received by a node in the mesh [8,9]. The need to keep nodes listening for incoming packets places a severe burden on the stationary mesh’s energy budget; such persistent power consumption requires bigger, heavier batteries on each node. In a scenario where MAVs must deploy the multihop WSN mesh in an unknown environment, fewer nodes can then fit in the payload, which in turn reduces the maximum communication range of the MAV.

Power efficiency has been addressed by medium access control (MAC) layer protocols for stationary wireless networking stacks. By scheduling wireless communications, nodes can power down their radios entirely when not in use. These time division multiple access (TDMA) schemes allow duty cycling on the order of of 0.1%–1% or less [10–13], significantly lowering the average power consumption and battery requirement of each node. These protocols can also employ frequency diversity, scheduling successive communications on different frequency channels, thus mitigating effects of radio interference. While such power efficiency and reliability has been addressed by medium access control (MAC) layer protocols in stationary wireless networking stacks, these energy efficient MAC layers have until now not been extended to include mobile nodes.

Since line-of-sight communication can not be assumed, and current low-power WSN architectures are not suitable for MAV missions, a new architecture must be proposed to ensure low power consumption and communication reliability, while permitting rapid, high throughput data bursts when required.

### 2.3. Sample Hardware

**Figure 2.**
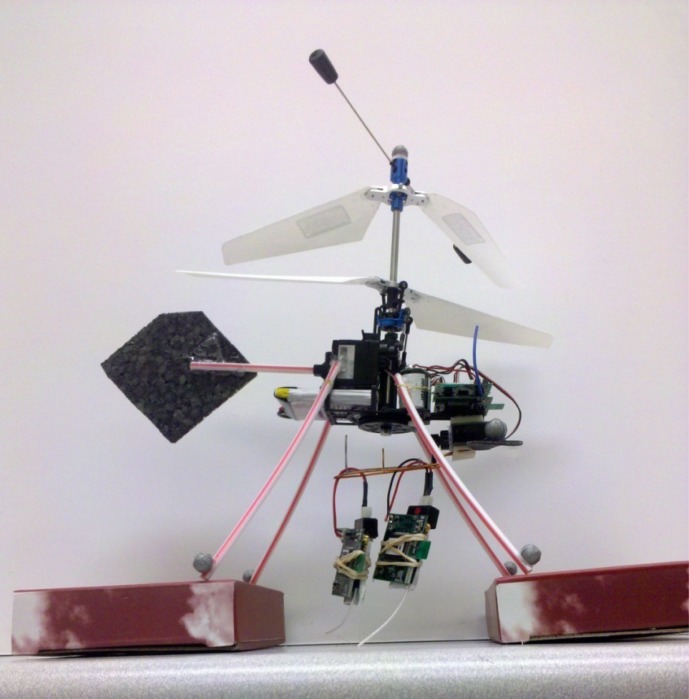
GINA hardware is used to implement an integrated MAV + WSN system.

Our MAV platform combines a miniature helicopter with an ultra-low-powered wireless node (Figure 1). The GINA board [14] consists of a 2.4 GHz IEEE 802.15.4 radio, Texas Instruments MSP430 microprocessor, and a number of inertial sensors. The GINA platform can be used directly as a MAV flight controller, or as a relay for control signals from a central location [15]. The OpenWSN project [16] has been ported to the GINA hardware to implement time synchronization, frequency channel hopping, mesh networking, and multihop communications. A MAV controlled by a GINA can be used to deploy additional GINA nodes for the stationary drop-off mesh infrastructure, as shown in Figure 2. With standards compliant networking interfaces, this hardware forms a testbed for the implementation of the protocols described in this paper.

## 3. MAV Communication

A MAV interacts with its communication system in two major ways, which are tied to its two major functional subsystems: flight and sensing.

### 3.1. Flight

Different levels of autonomy require different amounts of communication. If the on-board controller is solely responsible for stability, then a human operator must remote-control the flight. In that case, the base station needs to obtain sensor data of sufficient fidelity to understand the environment in order to direct the MAV. This is generally at least video at a few frames per second, perhaps augmented with additional inertial sensor data. This typically requires a throughput on the order of 100kbps. The operator must also send control signals back to the MAV. This is generally a much smaller amount of data, on the order of tens to hundreds of bits per second.

This bidirectional data needs to be transmitted in real time and minimizing latency is key. Without intelligence on board the MAV, a network delay on the order of tenths of a second could drive the MAV into a wall or obstacle. Slower, more stable MAVs may be able to tolerate the delay, but may waste energy in low speed movement. As MAV functionality is optimized, round trip latency becomes even more critical.

The required data rates and latencies are typically not difficult to achieve in a point-to-point link, and can even be attained over a short multihop path. However, the performance rapidly degrades as the distance between the MAV and its base station increases. Each additional hop along a network path adds both latency and network traffic, and as network traffic increases, the constant total bandwidth results in lowered end-to-end data throughput.

This saturation can be alleviated by requiring autonomy of the MAV. This can range from merely obstacle avoidance along a user-defined waypointed path to full autonomous path planning and guidance decisions. Autonomy thus minimizes or eliminates intervention from a human operator, and similarly, reduces the data that then needs to be sent to the base station for flight control. Nonetheless, data throughput and latency are still important parameters that impact the ability of human operators to influence the MAV mission.

### 3.2. Sensing

MAVs necessarily carry a number of sensors. At minimum, these include sensors for stability (e.g., inertial sensors, laser range-finders, *etc*.), but additional sensors may fall into one or more categories:

- Sensors that require significant mobility, e.g., still and video cameras;
- Sensors that are particularly heavy, e.g., chemical sensors;
- Sensors that need to be used only once per location, e.g., thermal sensors, passive infrared (PIR) sensors.

Many of these, such as thermal or chemical sensors, generate data at low rates, on the order of a few bytes per sample. Also, since the measurements often lack high spatial or temporal variability, samples are infrequently generated. In this case, throughput is of minimal concern. If the gathered data does not impact the real time mission status, then latency requirements can also be relaxed.

Other sensors, however, can generate large volumes of data. Of these probably the most widely applicable and thus most common are cameras, which can generate anywhere from kilobytes to megabytes per sample, at rates from minutes per sample up to samples per second. High latency can affect the throughput if data flowing through the network saturates node capacities, thus resulting in dropped data. If the gathered data does not critically impact the real-time mission, latency may not be so much of a concern, but maximizing data throughput still remains critical. It is thus imperative that the network containing the MAV support both rapid data streams, and well as more sporadic, smaller sized packets.

## 4. Stationary Sensor Network

### 4.1. Low Power Persistent Sensing

Reliable communication with mobile MAV agents demands a stationary wireless network, which routes information to and from MAV agents, while serving as a persistent sensing platform to process information about its surrounding environment. While MAV agents are deployed for short-term mission-specific purposes, this stationary network is expected to have deployment lifetimes on the order of days to months. As such, low power consumption is key for maximizing battery and deployment lifetime. Compared to the requirements imposed by mobile MAV agents, the data throughput requirements of stationary network nodes are expected to be low, generating packets on the order of seconds to minutes. For example, in mission critical settings the stationary network would be responsible for environmental-sensing tasks such as proximity sensing or footstep detection.

### 4.2. Wireless Sensor Networks

Some notable studies have explored the use of multihop WSNs for purposes of long-term persistent sensing. A number of such systems are time division multiple access (TDMA) based networks which support both time and frequency division to facilitate robust communications while permitting for low battery consumption [13,17]. Successful applications have ranged from industrial control [18], military applications [19], and habitat monitoring [20]. The majority of these studies was built upon the IEEE 802.15.4 stack [21], which specifies hardware and software requirements to be met to facilitate interoperability between low-power wireless devices.

A number of key factors emerge when considering the needs imposed on the stationary communications infrastructure:

- Reliability: Guarantees must be given to ensure reliable data packet delivery, and the network should have methods to mitigate the effects external radio interference.
- Low power consumption: Stationary nodes have to meet long-term deployment goals while running only on batteries.
- Scalability: The network should support an arbitrary number of stationary or mobile nodes.
- Security: Communication should be encrypted to protect data integrity and mitigate malicious attacks.
- Flexibility: The communication protocol has to dynamically allocate resources to meet the needs imposed by high data throughput requirement of MAVs with those of the low throughput stationary sensing nodes.

To meet these requirements for the stationary network backbone, we propose an architecture built upon the time synchronized mesh protocol (TSMP) [13]. This protocol has also been standardized under the IEEE 802.15.4E workgroup [22], as well as the Wireless Hart Foundation [23], and ISA100 [24]. At its core, it ensures reliable, secure, and scalable wireless communications by combining very tight time synchronization with frequency channel hopping and routing diversity. These protocols also specify authentication and encryption routines.

**Figure 3.**
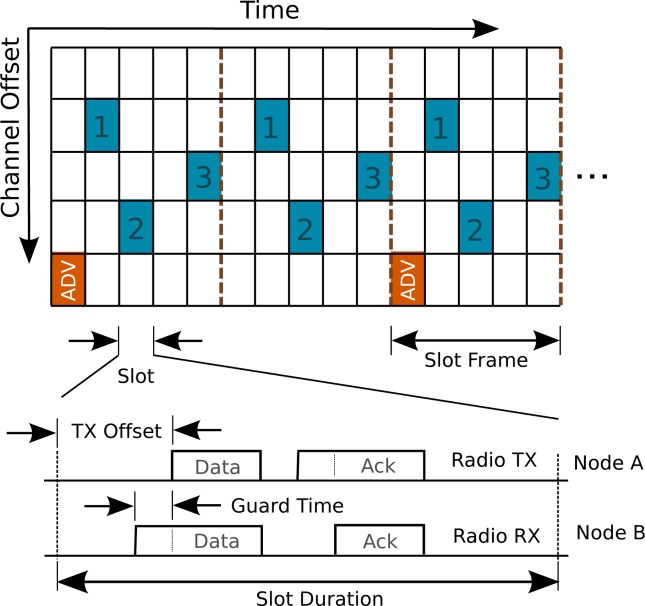
Time synchronized channel hopping in WSNs: a node synchronizes to its time parent by listening to advertisement (ADV) packets. Packets are sent a predetermined time into a slot, permitting for efficient resynchronization. Once synchronized, a node is assigned a slot and channel offset, and only communicates with neighbors according to this schedule.

TSMP relies on a number of MAC-layer enhancements (and thus larger implementation overhead) to facilitate synchronization and frequency channel diversity. It is a networking technique that relies on an agreed upon transmission schedule between network nodes (Figure 3). Time is sliced up into time slots of equal length (10ms according to the IEEE 802.15.4e standard); a constant number of slots make up a slot frame which repeats indefinitely over time. Once synchronized, network node pairs are scheduled to exchange communications at a specified time slot and channel offset (carrier frequency, 16 of which are specified by IEEE 802.15.4) within the repeating slot frame. A major requirement stipulates that no two node pairs can communicate during the same time slot and on the channel offset, thus ensuring collision-free communication. It has been shown that multipath fading and external interference can drastically impede network connectivity if a single frequency channel is used [7]. Since nodes only communicate when scheduled, they keep their radios powered off most of the time. This low duty cycle plays a critical role in keeping battery consumption to a minimum, theoretically permitting one of our nodes to last years on a standard AA battery. Such a TDMA-based network can significantly increase the throughput of a WSN, while reducing energy consumption and packet collision rates [13].

The workgroup IEEE 802.15.4E [22] focuses on enhancing the MAC protocol proposed in IEEE 802.15.4 while keeping the same physical layer. In its current proposal, nodes can switch between different hopping sequences. As with TSMP, slots can be added/removed during the lifetime of the network.

In the stationary setting, a TSMP network is centrally scheduled by a network manager. When a node first joins a network, it leaves its radio on and listens for advertisements (ADV packets, see Figure 3) from its neighbors. Upon hearing an ADV packet, the node synchronizes to the network by the adjusting its slot frame to reflect that of its time parent. The network manager than assigns this new node a transmission schedule. The network manager may allocate more slots to a node to increase throughput. Due to clock drift, nodes are also required to re-synchronize regularly. The joining and synchronization process may take some time, depending on the size of the network, external interference, and the length of the slot frame. As such, a traditional TSMP network does not lend itself readily to applications which require mobile agents and fast response times. The TSMP routing table, and transmission schedule are updated at most once every slot-frame. A MAV agent may change its location, and thus its neighbors, much more quickly than a centralized controller can keep track off. As such, a modification must be made to the TDMA-based network schedule to accommodate the dynamics of the MAV.

Aside from supporting scheduled communications between neighboring nodes, TSMP also supports multihop communications via the Routing Protocol for Low power and Lossy Networks (RPL) [25]. RPL is a gradient-based routing algorithm which provides multiple data paths between nodes. Data flows, one hop at a time from one node to another. This permits nodes that would otherwise be out of range to communicate with each other. Detailed information on such routing is given in [13,25]. While TSMP architecture is designed to facilitate low throughput communications, it can be augmented to allow sporadic bursts of large data as shown in the next section.

## 5. Hybrid Network

Table 1 shows the significant differences in design parameters which are imposed when comparing stationary nodes to MAV nodes.

Rather than building a single network architecture that compromises on all these metrics, we propose instead to have a dual-mode network. The deployed stationary network operates by default as a standard TDMA-based mesh. As new nodes are dropped off by the MAV, they join the mesh and carry on as described by TSMP standards. A modification is added to facilitate communications with the MAV; this communication is carried out on an independent set of channels. In an IEEE 802.15.4 network, 16 channels are typically available for transmission. In the proposed architecture, a subset of these channels is reserved for low throughput stationary mesh communications, while the remainder is only used when required by MAV agents.

### 5.1. TSMP Modification

The stationary network dropped off by the MAV implements a low power, low duty cycle TSMP mesh. As nodes are dropped off, they join that mesh, with the routing configurations updating accordingly. The frame length must initially be set short enough to incorporate new nodes into the network on a rate comparable to the deployment of the nodes by the MAV. After the MAV mission, this can be slowed down as necessary.

Because the MAV is highly mobile, it will not maintain a constant set of neighbors, and so it is not used as a routing node in the mesh. It still listens for advertisement (ADV) packets to maintain synchronization with the network, but never sends out its own ADVs. Instead, it can send an acknowledgment packet (ACK) to an ADV to initiate communication into the network. After sending an ADV, stationary nodes listen to receive the ACK from a MAV. The advertisements are scheduled inversely proportional to neighbor count, so that on average the MAV would be able to hear one ADV every frame within a neighborhood without risking packet collisions. The frame length must be short enough so that the MAV remains within range of at least one node for the duration of a frame.

An extra slot is added to the frame after the ADV slot for communication from the network to the MAVs. If a node received an ACK to its ADV in the previous slot, it can then confirm with the MAV during the following MAV slot that it will switch to MAV mode for the duration of that frame. The proposed method is very similar to a slotted ALOHA scheme [26]. If two MAVs respond to an ADV at the same time, a packet collision may occur. In this case, a randomized back-off timer is used to retry communication at a later time. Once a MAV receives a clear-to-send signal during the second slot of each frame (ACK slot) it enters one of two throughput modes.

### 5.2. Low Throughput MAV Mode

**Figure 4.**
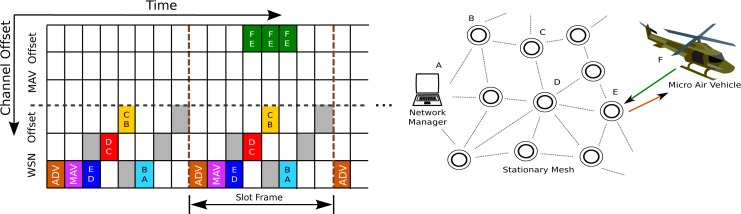
If the MAV has a small amount of data to send, it can request a number of slots to communicate to its nearest neighbor in the mesh. These slots can be predetermined by the manager or stationary nodes. That data then gets routed over the mesh as would any other data generated in the mesh.

When ACKing an ADV packet, the MAV specifies the type of data it is intending to transmit. If the the data to be transmitted can be fit into a few packets, without stringent latency requirements, the mobile node can send this over the reserved MAV channels to the stationary node in the mesh. The stationary node responds to the MAV with slots in the frame during which it can receive data from the MAV. The MAV then communicates with the mesh as would any other node during its scheduled slots, and the resulting packets can then be routed through the stationary network according to the existing transmission schedule. This mode is outlined in Figure 4. Multiple MAVs can simultaneously communicate to the mesh in this mode.

### 5.3. MAV Burst Mode

**Figure 5.**
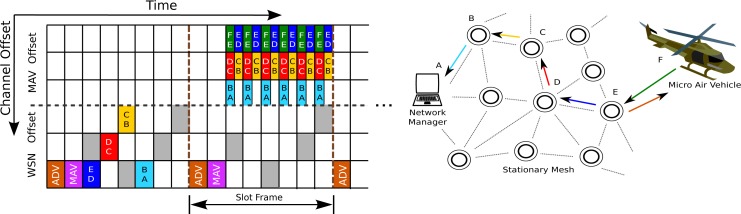
In burst mode, the nodes along the direct path from the MAV to the base station remove themselves from the TSMP network and communicate in a separate high throughput, low latency mode.

When the MAV has high volumes of data to transmit, or when latency is a primary concern, sending packets through existing mesh traffic becomes insufficient. In this case, the MAV requests a network mode switch in its ADV ACK. If this is confirmed by the stationary node in the MAV slot (see Figure 5), then a dedicated multihop route will be opened up between the MAV and the base station. This route from MAV to base station is determined during standard operation of the mesh, e.g., as described in [10], and is unlikely to change over the duration of a slot-frame. Starting from the node in contact with the MAV, each node in the mesh informs its direct parent of the mode switch, and then shifts to the burst mode.

This upstream notification can happen in two ways. The standard TSMP communication could be used, in which case the notification would reliably propagate upstream over the course of a slot-frame. Burst mode could then be executed in the following slot frame. Alternately and preferably, a separate slot following the MAV slot could be dedicated to this function, in which every node would listen for a mode-switch notification, and immediately switch to burst mode if required.

The burst mode communication happens over an independent set of channels from the main mesh. During the streaming mode slot-frame, nodes not involved in the direct path from the MAV to the base station carry on network operations as usual. Meanwhile, those nodes directly in-route between the MAV and base station establish a separate communications schedule over their channels. While burst communication schedule is similar to the standard TSMP communication, there are a number of important differences.

Since the data is a single, unidirectional, burst mode stream over a predefined path, most of the TSMP headers can be stripped out of the packets to yield a higher data throughput. For similar reasons, the slot length can be narrowed to transmit at maximum radio output (about 5ms per slot in an IEEE 802.15.4E setting) to facilitate higher packet density. For a standard IEEE 802.15.4E network, this can have the effect of doubling the number of available transmission slots. Link level ACKs are also eschewed in favor of higher bandwidth as this sort of data typically does not require very high reliability. This mode is shown in Figure 5.

This burst mode subnetwork persists for one slot-frame. On the next frame, the network returns to its original state. Data still in transit along the path can either be discarded or stored for transmission over the default channels. If the MAV has additional data it needs to transmit, it can again request burst mode operations by responding to the next ADV packet it receives.

## 6. Analysis and Conclusions

### 6.1. Overview

Extensive analysis on TDMA networking, in particular TSMP, has demonstrated its importance for low power wireless networking [7,13,27]. The low throughput MAV mode is effectively just an extension to a standard IEEE 802.15.4E (TSMP) network, with out-of-band communication reserved for direct point-to-point communication with the mobile MAV. Network maintenance and the remaining traffic is left to the primary unmodified IEEE 802.15.4 mesh, thus ensuring the robustness, reliability, and scalability inherited from the original standard. This time synchronized communication protocol has been implemented on the GINA motes under the OpenWSN stack, validating 10ms slots with an extremely efficient duty cycle for the stationary mesh [16]. This stack supports channel hopping and multihop routing at very low power.

Burst mode communications, on the other hand, augment the underlying TSMP MAC layer with an additional independent set of channels for the MAV burst data. This additional network is effectively a separate, stripped down TSMP network, derived from the original mesh but operating on a separate set of channels. Given a single unique path from a MAV to the base station, a MAV cannot simply send one packet after the other. Since its parent node must forward the packet, the MAV must pause until the parent node is ready to receive again. This leads to the transmission schedule seen in Figure 5.

### 6.2. Tuning Network Parameters

The hybrid network requires partitioning the available network channels between the stationary mesh and MAV communications. With one unique path to the base station, the optimal scenario involves the ability of the MAV to transmit a packet every other slot. If not enough slot offsets are available, the paths to the base station will fill the transmission schedule in a way that does not permit the MAV to transmit a packet every other slot. This is considered sub-optimal.

**Figure 6.**
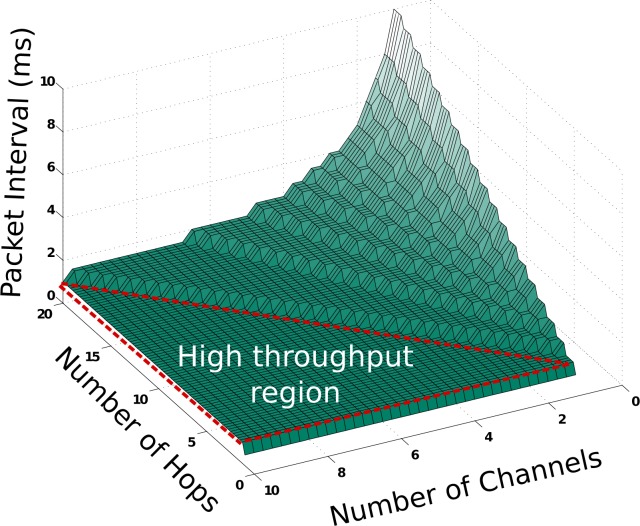
Given the expected maximum network distance (number of hops) from the MAV to the base station, a number of channels can be allocated to the MAV burst mode. As long as the number of channels is at least half the number of hops, the MAV will be able to communicate at the maximum supported throughput of the network given one unique path to the base station.

As a rule of thumb, the number of hops (paths to the base station) can only be twice the size of the reserved channels to ensure that the MAV can transmit a packet every other slot. As a MAV gets more hops away from the base station, more channels should be allocated accordingly to ensure that burst mode is carried out at the fastest possible rate. As such, to achieve high throughput, the number of allocated MAV channels must be at least half the number of hops that the data is expected to traverse (see Figure 6).

With half-duplex radios, each node along the route must spend half its time receiving and half its time retransmitting the data along the pipe, thus limiting the throughput to half the available data bandwidth. However, with a sufficiently dense network, this limitation could be lifted. If two completely independent paths can be found from the MAV to the base station without overlapping intermediate nodes, data can be alternately sent along both routes, further doubling the throughput to the full available data rate. In this case, the number of reserved MAV channels would need to be at least half the number of hops from MAV to base station.

As there are only 16 channels available in the IEEE 802.15.4 standard, allowing a more extensive network requires more channels to be allocated for MAV use, and fewer for the underlying stationary mesh. However, as shown in [27], the robustness and efficiency benefits of channel hopping become evident with only a small subset of channels; peak performance can be achieved by allocating six channels to the stationary mesh. Ten channels can thus be available for MAV communications, allowing for a reliable network up to 20 hops deep.

### 6.3. Performance Evaluation

**Figure 7.**
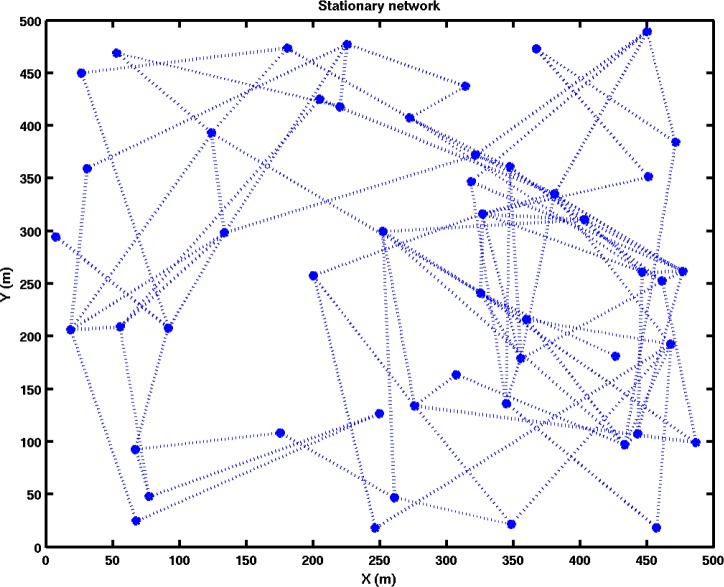
A simulated environment has stationary network nodes through which a MAV may travel. Network connectivity is indicated by dotted lines. Note that due to the nature of RF communication, connectivity is not geographical. Similarly, as the MAV travels around the network, the node to which it can communicate may change.

To evaluate the feasibility of extending a TSMP network to communicate with a mobile agent, a sample network was simulated using MATLAB. An area was populated with randomly distributed network nodes at varying densities (see e.g., Figure 7) through which moved the mobile node. Time varying connectivity between nodes was modeled using a stochastic noise penalty atop the Friis equation [28]. This allows for the empirically observable random packet loss despite high average signal strength.

Under the proposed scheme, the MAV communicates into the mesh during a slot frame in which it receives an ADV packet from a nearby stationary node. If the stationary nodes send ADVs too infrequently, the MAV may not receive any packets; on the other hand, if the nodes transmit too often, the MAV may encounter a packet collision, again resulting in a failed ADV. The optimum is reached when each node transmits an ADV with probability inversely proportional to the number of neighbors it can hear.

**Figure 8.**
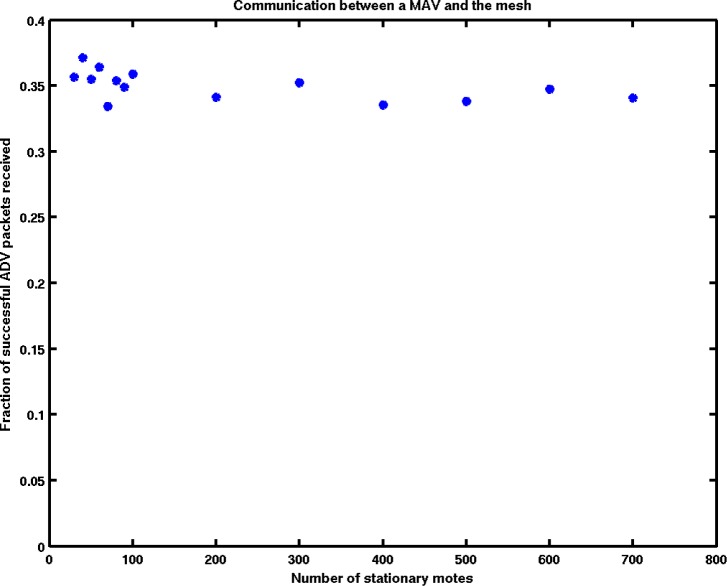
Regardless of the density of the underlying mesh network, the MAV successfully hears a fraction of the advertisement packets, and so can communicate during approximately 35% of the available slot frames.

In that case, and accounting for stochastic packet loss, the MAV received a successful ADV ≈ 35% of the time, regardless of density (see Figure 8). In those slot frames, then, data can be offloaded from the MAV into the mesh. Once in the mesh, the data can be transmitted to the base station as governed by the TSMP network parameters [13]. Depending on the desired metric, dropped data packets can be retransmitted to ensure reliability, or ignored to preserve latency.

This is less frequent than can be achieved with a single hop always-on point-to-point link. However, this scheme is inherently robust to scaling. It allows for reliable multi-hop communication even as the position of the MAV and the network topology change. The underlying TSMP network also eliminates the problem of network saturation due to broadcast flooding: since each packet transmission is scheduled and routed, the number of packets does not increase exponentially as the MAV gets farther from the base station. In that regard then, the reduced throughput is necessary to ensure that the MAV data can arrive at the base station.

## 7. Conclusions

This paper proposed a communication infrastructure to support highly mobile MAV nodes in a stationary low-power mesh. In contrast to earlier research, simulations have shown that it is feasible to extend a TSMP-like WSN stack, which features an IEEE 802.15.4E time synchronized channel hopping MAC layer, to enable rapid, reliable, low power multihop communications with mobile agents.

Our architecture reserves a unique set of channel offsets to MAV agents, and permits for both low-throughput and streaming data to be transmitted by the MAVs. This flexibility comes at no cost to network reliability, as effects due to external and intra-network radio interference are mitigated through scheduled communications and channel hopping. Furthermore, battery resources of the stationary mesh are preserved due to an extremely low radio duty cycle. This system proposes to greatly increase the range and duration of MAV missions in RF denied environments; future work will focus on extending the already implemented stationary communication infrastructure for the GINA [16] with our proposed hybrid architecture to gather experimental performance metrics.

## Figures and Tables

**Table 1.** Differences in design parameters between stationary and mobile nodes in a hybrid sensor network.

**Metric**	**Stationary network**	**MAV nodes**
Duration	10^6^ seconds	10^3^ seconds
Data rates	10^2^ bits/second	10^5^ bits/ second
Latency	10^2^ seconds	10^−2^ seconds
Network routing	stable	dynamic
